# Medicinal Virtues of Warm Milk

**Published:** 1884-07

**Authors:** 


### MEDICINAL VIRTUES IN WARM MILK.

IT is stated that warm milk is a valuable remedial agent in certain diseases. In the East Indies warm milk is used to a great extent as a specific for diarrhœa. A pint every four hours will check the most violent diarrhœa, stomach-ache, incipient cholera, and dysentery. The milk should never be boiled, but only sufficiently to be agreeably warm, not too hot to drink. Milk which has been boiled is unfit for use. A person who has had extensive experience says it has never failed in curing in six or twelve hours, and I have tried it, I should think, 50 times. I have also given it to a dying man who had been subject to dysentery eight months, latterly accompanied by one continued diarrhœa, and it acted on him like a charm. In two days his diarrhœa was gone; in three weeks he became a hale, hearty man; and now nothing that may hereafter occur will shake his faith in hot milk. A writer has also communicated to the *Medical Times* and *Gazette* a statement of the value of milk in 26 cases of typhoid fever, in every one of which its great value was apparent. It checks diarrhœa, and nourishes and cools the body. People suffering from diseases need food quite as much as those in health, and much more so in certain diseases where there is a rapid waste of the system. Frequently all ordinary food, in certain diseases, is rejected by the stomach, and even loathed by the patient; but nature ever beneficent has furnished food that in all diseases is beneficial ; some directly curative. Such food is milk. Dr. Alexander Yale, after giving particular observations on the point above mentioned, its action in checking diarrhœa, its nourishing properties, and its action in soothing the body, says: "We believe that milk nourishes in fever, promotes sleep, wards off delirium, soothes the intestines, and, in fine, is the *sine qua non* in typhoid fever." [The use of fresh sweet milk, as above suggested, is good for children; but the adult stomach digests milk far more readily when it is converted into "koumiss."—Ed Journal of Health.]

